# Enabling active and healthy ageing decision support systems with the smart collection of TV usage patterns

**DOI:** 10.1049/htl.2015.0056

**Published:** 2016-03-23

**Authors:** Antonis S. Billis, Asterios Batziakas, Charalampos Bratsas, Marianna S. Tsatali, Maria Karagianni, Panagiotis D. Bamidis

**Affiliations:** 1 Laboratory of Medical Physics, Medical School, Aristotle University of Thessaloniki, 54 124 Thessaloniki, Greece; 2 School of Mathematics, Aristotle University of Thessaloniki, 54 124 Thessaloniki, Greece; 3 Open Knowledge Foundation Greece, 54 124 Thessaloniki, Greece

**Keywords:** geriatrics, home computing, ubiquitous computing, patient monitoring, decision support systems, medical expert systems, TV usage patterns, behavioural patterns, daily living activities, smart decision support systems, health smart homes, smart monitoring sensors, inexpensive monitoring sensors, unobtrusive monitoring sensors, software tools, smart monitoring system, remote monitoring system, mental health change

## Abstract

Smart monitoring of seniors behavioural patterns and more specifically activities of daily living have attracted immense research interest in recent years. Development of smart decision support systems to support the promotion of health smart homes has also emerged taking advantage of the plethora of smart, inexpensive and unobtrusive monitoring sensors, devices and software tools. To this end, a smart monitoring system has been used in order to extract meaningful information about television (TV) usage patterns and subsequently associate them with clinical findings of experts. The smart TV operating state remote monitoring system was installed in four elderly women homes and gathered data for more than 11 months. Results suggest that TV daily usage (time the TV is turned on) can predict mental health change. Conclusively, the authors suggest that collection of smart device usage patterns could strengthen the inference capabilities of existing health DSSs applied in uncontrolled settings such as real senior homes.

## 1 Introduction

Demographic changes caused by the increase in life expectancy are a reality, especially in Europe [1], and have shifted research focus to the study of seniors’ physical and mental health. Ageing can have negative consequences to the mental-and-physical health of the elderly due to natural cognitive and physical decline but also due to other co-morbidities, chronic diseases and social isolation.

Decision support systems (DSSs) have been widely used in the field of clinical practice in order to assist healthcare professionals to analyse data, make decisions and take appropriate actions in the health management of their patients. Data-driven and knowledge-driven approaches are the two mainstream methodologies to develop a DSS. The former approach has emerged lately within ambient assisted living research field due to the plethora of data produced by off-the-shelf sensors and devices and their openness for further processing and analysis.

Continuous recording of health and lifestyle parameters anywhere/everywhere or else ubiquitously or ‘in the wild’ allow for more accurate and detailed representation of subtle changes that might be early signs of health deterioration. Real-time decision support based on ubiquitous computing drive the change toward a patient-centred decision making process.

Everyday life activities, as they are represented through basic activities of daily living and instrumental activities of daily living are considered as the best practice for practitioners to recognise health deterioration risks [2]. One of the main topics in machine learning and decision support literature concerns the effective classification and recognition of ADLs [3–5] and deviation detection from routine patterns [6]. Once ADLs are modelled, routine patterns are modelled based on the sequence of activities and duration of each activity within the routine. Hidden Markov models (HMMs) appear to be the most successful technique to characterise routines in ADL [7, 8]. Accurate classification of ADLs is the first step for identifying changes in the routine habits of the elderly to determine their health conditions. Deviation characteristics such as activity duration may prognose that an irregular activity occurred.

Another trend in ADL literature is its quantification through sensor systems, based on widely accepted clinical assessment tests. Sensor readings such as walking speed or location features are correlated to battery tests such as assessment of motor and process skills [9]. Longitudinal data analysis has also been used to correlate changes in co-morbidities and functional assessment tests to irregularity and dissimilarity in sensor data observations [10]. However, these approaches focus mainly in visualising sensor patterns, rather than applying any data mining or statistical analysis of scored ADLs.

One of the causes that lead to seniors’ health problems and can be represented through ADLs has been sedentary lifestyle: in fact seniors spent most of their awake time doing sedentary activities [11]. Inactivity in late life can be translated into activities such as TV watching. Many research programmes have been investigating the relationship among the TV watching habits of the elderly and their mental-and-physical health. A large number of studies have found links between TV viewing time and various physical health problems of older adults [11, 12]. For example, in the European prospective investigation of cancer study 13,197 elderly were followed-up for death ascertain, showing a high association between the increase of TV watch time and an increased risk of mortality caused by cardiovascular issues [13].

However, sedentary behaviours have been shown to affect the mental health of seniors as well. The English Longitudinal Study of Ageing conducted a 2 year follow-up of 6090 community-dwelling older adults in order to measure changes in TV viewing time between baseline and follow-up time points. Factors such as socioeconomic status, depressive symptoms, disability, chronic illness and physical activity were used as predictors of TV viewing time changes. Results showed that more TV viewing time is associated to lower socioeconomic status, presence of depressive symptoms and physical inactivity among others [14]. The same study showed the existence of evidence at baseline about self-reported TV viewing time correlation with higher depressive symptoms and poorer global cognitive function. However, other sedentary behaviours such as Internet usage affected mental health in a positive way [15].

In an Australian Diabetes, Obesity and Lifestyle study, quality of life subcomponents (physical, mental and vitality summary sub-scores) were associated to TV viewing time (hours per day) in a large cohort of Australian adults. Main outcomes of the study were summarised as for the negative consequences of the TV viewing time with respect to physical and mental well-being [16].

Da Ronch [17], has analysed data from 1383 seniors, as part of an international research project called MentDis_ICF65+ and found that there are associations among TV watching and various mental health issues such as cognitive impairment, demoralisation and depression.

Researchers have implemented various methods in order to remotely retain information about at home television usage of seniors. A feasibility study was conducted by Nakajima *et al.* [18] in order to monitor TV operating state. They extracted 1 year patterns of TV watching time by developing an inexpensive sensor and attaching it to the power line of the TV set. This system was then retested with added functions that allowed continuous remote monitoring and storage of data through an Internet connection [19].

Tsukamoto *et al.* [20] evaluated a monitoring system that consisted by electric field sensors. While this system can be used to measure the usage of various household electronic devices, they report accurate measuring of television usage. Shen *et al.* [21] have developed a low-cost embedded multimedia terminal in order to turn a conventional TV to a smart communication channel. This extended TV device was able to gather information related to TV watching such as the frequency of turning on/off TV (times per day), the time length of each watching TV (minutes) and the used watching channel (quantity per day). These features were used as input to machine learning algorithms such as support vector machines and artificial neural networks in order to derive psychological status of elderly people. Results obtained were described as very accurate and promising. Suryadevara *et al.* [22] measured TV usage through wireless sensing units that were extension to the devices power cord. This was done as part of research done in a smart home, where data was consequently used to predict wellness of elderly living.

However, previous attempts to monitor TV watching patterns require that elderly living environments need a retrofit. Seniors tend to reject technology when it becomes an impediment in the home environment or aesthetically incongruent [23]. In this Letter, we build on top of the literature findings and describe an unobtrusive way to remote monitor TV usage patterns in the living environment of four senior lone-living women, without the development of any external, bulky devices or sensors. On the basis of long-term monitoring of TV operating status, we have managed to identify statistical correlations between measured TV usage time and mental health trends. In the remainder of this Letter, we provide a detailed description about the smart collection and management of TV operating state data, the analysis performed to extract change points and in particular their direction and the presentation of statistically significant correlations among sensor data and ground-truth assessment records provided by experts. Finally, we shed light on the current research work on the field along with research limitations and further envisaged work.

## 2 Smart TV monitoring system

The TV operating state monitoring system consists of a smart TV and a custom developed software that runs on a personal computer (PC) and samples the TV operating state. TV and the PC that runs the software are on the same local area network.

Smart TVs have emerged recently due to their capability of offering advanced computing services and Internet connectivity. Apart from the conventional services offered by contemporary digital TV sets such as electronic program guides, smart TVs are computer systems that provide improved functionalities since they allow the installation of rich Internet applications. The broad range of applications delivered through a smart TV include among others: games, social networking, video communication, multimedia applications and voting. Philips 7000 series was delivered to seniors’ homes and connected to the Internet via the local area network as part of the unobtrusive smart environments for independent living system [24].

Seniors could access, apart from channels, apps via a press on the button on the remote control as well. While watching TV, seniors could get to see the smart TV portal filled with apps with a press on the button on the remote control and choose an app with the remote controller.

**Fig. 1:**
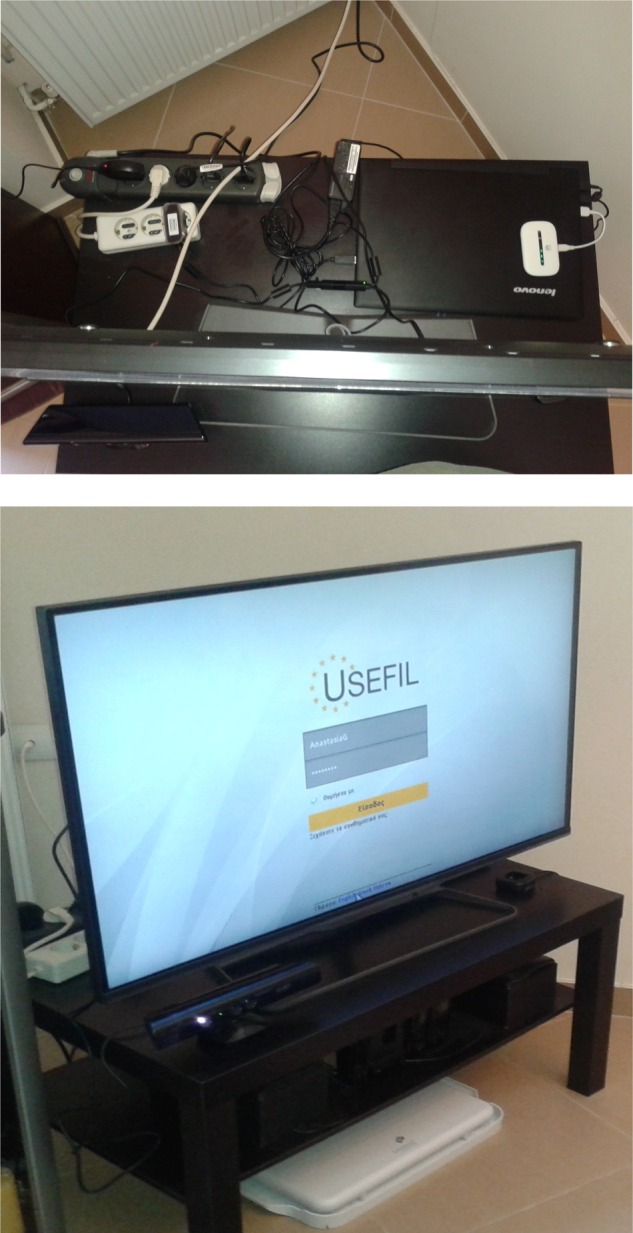
Smart TV monitoring system setup top: laptop running Java monitoring app 24/7 plus connections; bottom: Philips smart TV

As mentioned, apart from the smart TV, a Java application run on a laptop, monitoring the TV usage status, e.g. whether TV is on or off, which channel is the user currently watching. The sampling rate was set every 5 min. Results were stored to a local resource description framework (RDF) database in the form of time stamped event observations, readily available for easy retrieval and further processing. A typical installation example is shown in Fig. 1.

## 3 User sample and data collection

Four elderly, lone-living women aged 75.5 ± 5.44 years and 15.5 ± 7.37 years of education were recruited. Three out of four seniors (3/4) had memory problems, while two of them had depressive symptomatology. Participants, that declared interest, were explained about the purposes of the home study, and on acceptance they signed an informed consent, declaring their voluntary participation. Seniors were examined by two experts at baseline and at several follow-up time points. Clinical staff administered a plethora of tests to assess global cognitive function (mini-mental state examination [25], montreal cognitive assessment [26], Trail A and Trail B [27]), socialisation (Friendship scale [28]), physical assessment tests (Fullerton Fitness [29] and Berg Scale [30]) and depression levels (patient health questionnaire (PHQ9) scale [31]).

All tests were conducted every two or three months, except for PHQ9 which was administered every 1 month approximately. Values of daily TV usage were gathered unobtrusively for 11 months by the Java app. The study was approved by the Bioethics Committee of the Medical School of the Aristotle University of Thessaloniki, Greece (Approval No. 93/26-06-2014).

## 4 Data processing

Event observations were retrieved from the SPARQL Protocol and RDF Query Language. All entries were inspected and duplicate measurements were deleted. Then, data were summed to obtain the total time TV usage per day in minutes and further erroneous values were identified and removed (hours per day >24). A synopsis of this data is given in Table 1.

**Table 1:** TV usage data and relevant health information for each participant

Subject	TV usage (number of days)	Mean time of usage ± standard deviation	Coefficient of variation, %	Memory problems	Depressive symptomatology	Number of PHQ/other test assessments
A	263	202 ± 151.4	74.7	yes	yes	6/4
B	275	389 ± 303.9	77.9	no	no	5/3
C	281	156 ± 142.2	91	yes	yes	5/3
D	78	432 ± 322.2	74.4	yes	no	3/2

**Fig. 2:**
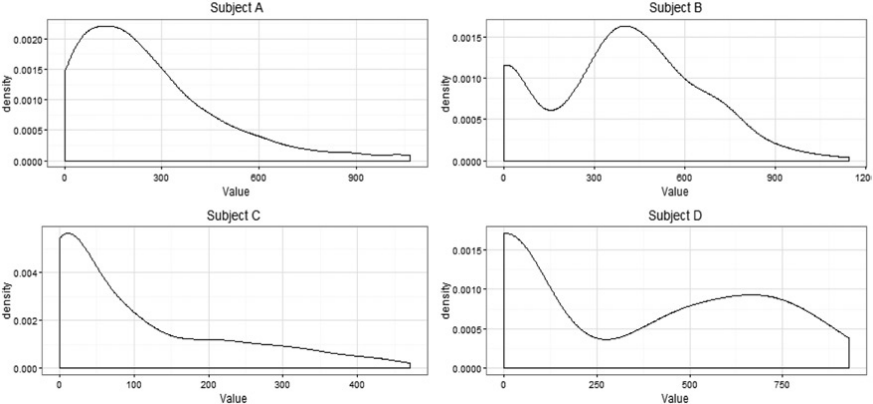
Distribution of the values of TV usage per subject via kernel density estimate

After preprocessing, the mean and the coefficient of variation were calculated for periods that differed in time for each test group. These periods were defined based on the date of the baseline or follow-up assessment: in the case of global cognitive function tests, quality of life assessment tests and the Friendship scale intervals lasted one month and more specifically ±15 days prior and after each test administration date point, whereas two week intervals, representing the weeks prior test administration, were chosen for the PHQ9 scale tests. Distributions of TV usage values per subject are depicted in Fig. 2. A Kruskal–Wallis *H* test showed that there was a statistically significant difference in TV usage values between the subjects, *H*(3) = 164.1, *p* < 0.001. Multilevel modelling (linear mixed effects models) [31] was used to test relationships between the time spent using TV and the various test scores. This type of modelling takes into consideration the within-subjects variance while allowing the exploration of relations between-subjects. *P*-values for statistical significance of the coefficients of the model were calculated using bootstrapping (*n* = 1000 simulations). Data processing and analysis was done with the R statistical software. All models are along formula (1), where *β* and *b* represent the fixed effects vector and the random effects vector, respectively

(1) $${\rm test}\, \, {\rm score} = {\beta } \times \, \, {\rm
              time}\, \, {\rm of}\, \, {\rm TV}\, \, {\rm usage} + {b} \times {\rm subject} + {\rm
              error}\eqno\lpar 1\rpar $$

## 5 Results

Various statistically significant correlations were found: there is positive correlation between the mean time of TV usage and PHQ1 test result (loss of interest), PHQ2 (depressive mood), PHQ3 test result (insomnia/hypersomnia) and PHQ sum. Detailed data is shown in Table 2. No statistically significant results were obtained for any other test scores.

**Table 2:** Estimates, errors and fit statistics for mixed effects models

Response variable	Effect	Estimate	Std. error	Pr > *t*	BIC
PHQ1	intercept	0.36197	0.36336	>0.05	56.9
	TV usage time	0.00115	0.00115	0.01^a^	
PHQ2	intercept	0.64390	0.35945	>0.05	48.0
	TV usage time	0.00304	0.001	0.01^a^	
PHQ3	intercept	0.064754	0.58324	>0.05	46.1
	TV usage time	0.003349	0.000868	0.003^a^	
PHQ sum	intercept	2.841025	1.9582	>0.05	104.7
	TV usage time	0.015087	0.004439	0.01^a^	

BIC: Bayes information criterion score.

^a^Statistical significance as *p* < 0.05.

## 6 Discussion

An unobtrusive system for the remote monitoring of TV usage patterns was developed, based on the importance of identifying daily rituals of the elderly people lives. Older people show an affinity for TV watching for various reasons such as companionship, information source, entertainment and establishment of daily rituals. TV usage patterns such as extended TV watching time, TV programme types and watching period time within day or night [20, 21]. On the basis of our preliminary analysis and results, there seems to be a significant correlation among TV usage time and mental health, as measured by experts. This means that by unobtrusively monitoring TV usage rituals, one may predict early signs of health deterioration. Previous attempts on remote monitoring TV usage patterns of the elderly equipped TVs with external, possibly bulky devices, in order to allow for the recognition of the TV's operating status, affect the unobtrusiveness of the end user system and ultimately their acceptance of installing into their living environments [23].

Future steps of this Letter include the use of additional information that we store and may better predict health deterioration such as time of the day that seniors watch TV, type of programme, e.g. comedy, drama and frequency of TV channels navigation. Apart from introducing additional features, we aim to apply more sophisticated data analysis based on existing decision support tools that has been previously published in [32]. Long-term trend analysis, based on statistical control processes or similarity and dissimilarity measures [33] could better visualise and identify abnormalities within daily routines of seniors’ lifestyles and more specifically their sedentary behaviour. Making this knowledge available to seniors through mHealth tools could help them to understand better any bad habits and empower them to change and follow better rituals, similar to what quantified self-movement [34] promotes.

A limitation of the current approach is that we took into account the TV usage time, not the actual time they watch TV, which might prove to be more accurate indicator for behavioural patterns shifts and abnormal events detection. However, this can be easily inferred based on the recognition of TV navigation, since we also have access to channel viewing of the user. Furthermore, due to very few data points (12) we could not find any relationships among TV usage patterns and global cognition or physical ability. Therefore, this result could not be considered as a safe conclusion and further data are needed to ascertain or reject any such assumption.

## 7 Conclusion

Seniors tend to follow certain rituals in their daily lives. One of the most known ritual is the TV watching. Underlying patterns have been correlated in the literature with physical inactivity level and consequently with health problems such as cardiovascular diseases. However, recently there are studies that showed that extended periods of TV watching are good predictors of emotional problems and mental decline. The work presented in this Letter, describes an unobtrusive TV operation status telemonitoring system for smart collection and management of TV telemetric data that would enable further decision support about early pathological signs. A longitudinal study was carried out in the homes’ of four participants for more than 11 months. Results suggest that TV watching time is related to depressive symptomatology such as loss of interest in daily activities, depressive mood, sleep deprivation and total depression levels. To this end, senior interaction with unobtrusive smart devices, e.g. smart TVs or tablets, could be seen as rich sources of information for patient-centred DSSs. The importance of supporting decisions with this kind of unobtrusive monitoring of daily living activities is believed to open up new ways of properly investigating cognition and its early symptoms of declination [29], as well as brain function in ecologically valid environments [30].
